# Severe heart failure in a unique case of cobalamin-C-deficiency resolved with LVAD implantation and subsequent heart transplantation

**DOI:** 10.1016/j.ymgmr.2024.101089

**Published:** 2024-05-07

**Authors:** Clara Hjalmarsson, Charlotte Backelin, Anders Thoren, Niklas Bergh, Jennifer L. Sloan, Irini Manoli, Charles P. Venditti, Göran Dellgren

**Affiliations:** a Department of Cardiology, Sahlgrenska University Hospital, Gothenburg, Sweden; b Sahlgrenska Academy, University of Gothenburg, Gothenburg, Sweden; c Department of Anaesthesiology and Intensive Care Medicine, Sahlgrenska University Hospital, Gothenburg, Sweden; d National Human Genome Research Institute, National Institutes of Health, Bethesda, USA; e Department of Cardiothoracic Surgery, Sahlgrenska University Hospital, Gothenburg, Sweden

**Keywords:** Cobalamin C deficiency, Heart failure, LVAD, Heart transplantation

## Abstract

Introduction

Cobalamin c deficiency (cblC), an inborn error of vitamin B12 metabolism, is caused by mutations of the MMACHC gene. It usually leads to a multisystemic disease; 50% of all patients with cblC have various structural heart defects. Severe congestive heart failure (HF) may also occur and its prognosis is poorly documented.

Case report

We present the case of a young man who had been diagnosed with cblC due to C331T mutation in the MMACHC gene at the age of 3 days and had been treated with substitution therapy (OH-Cbl, mecobalamine, carnitine, betaine, and calcium folinate) since then. He had mildly impaired cognitive function; an ectopic hypophysis/pituitary insufficiency, with adequate hormone replacement therapy; obstructive sleep apnea syndrome, treated with CPAP, bronchial asthma, and obesity (BMI of 30). The liver and kidney functions were normal. He developed severe dilated cardiomyopathy and HF at the age of 12y. With medical treatment, his condition improved and he was stable (NYHA class II) for several years. Six years later, his status deteriorated rapidly, as he developed advanced HF, INTERMACS 3. The cardiac ultrasound revealed dilated ventricles with severely depressed ejection fraction (EF), increased filling pressures, and pulmonary hypertension (sPAP 60 mmHg). Cardiac MRI showed extremely dilated chambers (LVedv 609 mL, RVedv 398 mL) with pronounced non-compaction, and a left ventricle EF of 13%. A primary prophylactic ICD and a left ventricular assist device (LVAD/HM3) were implanted, and the patient was subsequently listed for heart transplantation (HTx). After 25 months on the waiting list, he underwent an uncomplicated HTx. However postoperatively, he got two episodes of cardiac tamponade, as well as mediastinitis, treated with antibiotics and vaccum assisted closure. He developed severe kidney failure, which fully recovered after two months, and was treated successfully for an early moderate allograft rejection (ISHT 2). At the latest outward visit, twelve months after HTx, the patient was doing excellent.

Summary

To the best of our knowledge, this is the first ever reported case of a patient with CblC undergoing an LVAD implantation and subsequently a HTx. Although both interventions were complicated with bleeding events, this seems to be a treatment option for advanced HF in patients with CblC.

## 1 Background

Combined methylmalonic acidemia and homocystinuria cobalamin C (*cblC*) type (OMIM: 277400), an inborn error of vitamin B12 (cobalamin) metabolism, is caused most frequently by variants in the *MMACHC* gene [1,2]. Patients exhibit multisystem involvement with some afflicted with various structural heart defects. Left ventricular (LV) trabeculation, secundum atrial septal defect, dysplastic pulmonary valve, mitral valve prolapse, as well as pulmonary arterial hypertension [3]. Some late onset cases presenting with *cor pulmonale* show improvement with intensification of treatment [4]. Severe congestive heart failure (HF) in patients diagnosed early and treated with standard of care is rare [5].

## 2 Case presentation

We describe the case of a boy, from a consanguineous marriage, who debuted with neonatal seizures and was diagnosed at the age of 3 days with combined homocystinuria and methylmalonic aciduria, subsequently confirmed as a *MMACHC* c.331C > T, p.(Arg111Ter) homozygote. Trio exome sequencing revealed only the *MMACHC* variant, no other genetic mutations. He was treated with OH-cobalamin (1 mg/mL, initially 2 mg i.m., qd), methyl-cobalamin (5 mg/mL, 20 mg i.m., qd), carnitine (200 mg/mL, 2 g p.o., bid), betaine (2–3 g p.o., tid), and calcium folinate (15–20 mg p.o., qd), in agreement with the actual guidelines [6]. However, despite conventional management, the total homocysteine remained high. He developed severe dilated cardiomyopathy and HF at the age of 12y. By this time his weight was 79 kg and his height 168 cm (BMI 28 kg/m^2^). The serum concentrations of methylmalonic acid (MMA) and homocysteine were fluctuating between 42 and 69 and 76.8–130 μmol/L, respectively, while the methionine level was 23 μmol/L.

The left ventricle was dilated (LV end-diastolic diameter 74 mm, LV end-diastolic volume 230 ml) and had severely reduced systolic function, with an ejection fraction (EF) of 20–25%; the right ventricle (RV) was moderately dilated, with moderately reduced systolic function (TAPSE 13 mm). No signs of structural heart disease were noted, other then apical trabeculation. His clinical status improved but his HF never recovered; he remained stable (NYHA class II) for six years with medical treatment (Metoprolol, Spironolacton, Enalapril, Digoxin, and Furosemide). The NTproBNP decreased from 3340 ng/L initially to 500–800 ng/L. He also had obstructive sleep apnea syndrome, treated with CPAP, bronchial asthma, mildly impaired cognitive function, and an ectopic hypophysis/pituitary insufficiency, which was diagnosed around the age of 2 years, and was treated with adequate hormone replacement therapy (Somatropin 0.8 mg s.q., qd; Hydrocortisone 10 + 10 + 5 mg p.o., daily; Levothyroxine, 75–125 μg p.o., qd; Testosterone was added at the age of 16 years, at a dose of 750 mg s.q., once every third month). Despite referal to an obesity team, his BMI increased during the next five years to 32.6 kg/m^2^.

At the age of 18y, he developed palpitations and symtoms of advanced heart failure, INTERMACS 3 (Interagency Registry for Mechanically Assisted Circulatory Support), without an obvious trigger, and was admitted to the hospital.

**Fig. 1:**
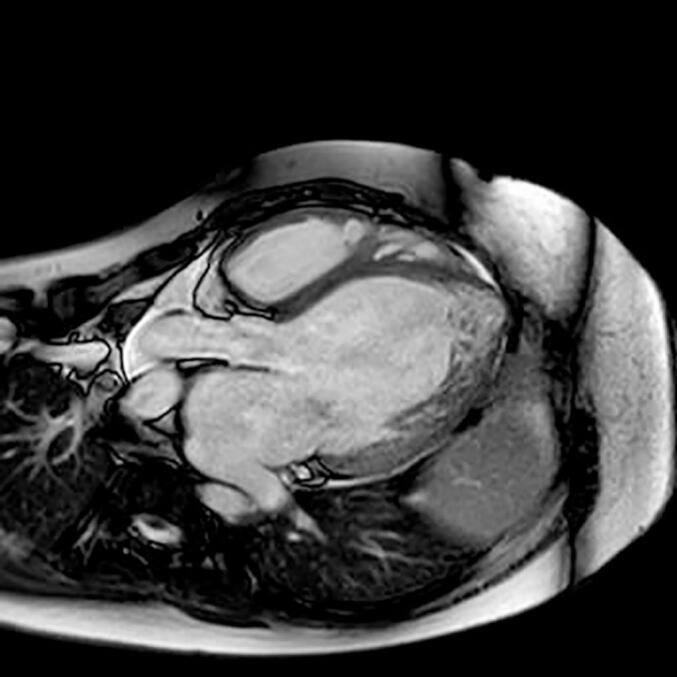
Cardiac MRI performed before the LVAD (HM3)-implantation showing markedly dilated left chamber with excessive trabeculation.

The ECG showed sinus rhythm with a heart rate of 90 bpm, bundle branch block with unspecific morphology and QRS-duration of 140 msec, and frequent premature ventricular contractions. The ultrasound of the heart revealed a dilated, hypokinetic left chamber with severe systolic dysfunction, elevated filling pressures, moderate mitral and tricuspid regurgitation, pulmonary hypertension (sPAP 60 mmHg); TAPSE 13 mm. A cardiac MRI (See Fig. 1.) showed dilated chambers with pronounced trabeculation, and systolic biventricular dysfunction (LVEF of 13%, LV end-diastolic volume 609 mL, LV stroke volume 77 mL; RVEF10%, RV end-diastolic volume 398 mL). There were no signs of end-organ damage, but the patient's status deteriorated rapidly. The serum concentrations of MMA and homocysteine were now 97 and 91 μmol/L. The dose of OH-cobalamin was increased to 30 mg qd. This higher dose was given i.v. due to the large volume needed, since a concentrated OH-cobalamin solution was not available in Sweden at the time. Right heart catheterization confirmed the impaired hemodynamics (cardiac index, CI 1.6 L/min xm^2^, mixed venous oxygen saturation, SvO2 50%), with moderate-severe post-capillary pulmonary hypertension (pulmonary vascular resistance, PVR 3 WU; mean pulmonary arterial pressure, mPAP 43 mmHg; pulmonary arterial wedge pressure, PAWP 31 mmHg). A primary prophylactic ICD and a left ventricular assist device (HM3) were implanted. During the early post-operative period after the implantation the patient suffered from bleeding complications, which were succesfully managed. He was subsequently listed for elective heart transplantation (HTx). Treatement according to general recommendations for patients with mechanical circulatory support, including optimal medical therapy for heart failure, and a strict metabolic control with iv OH-cobalamin was given throughout the bridging period. The serum concentrations of MMA, homocysteine, and methionine decreased after the latter dose adjustment to 8.1, 31.8 and 28 μmol/L, respectively.

After 25 months on the waiting list, he underwent an orthotopic heart transplantation (HTx). Postoperatively, he suffered from two bleeding episodes resulting in cardiac tamponade, as well as mediastinitis, and was treated with antibiotics and vaccum assisted closure. He developed severe kidney failure, which fully recovered after two months, and was treated successfully for an early moderate allograft rejection (ISHT 2R). At the latest clinic visit, 18 months after HTx, the patient's status was markedly improved. The graft function has, so far, not shown any signs of being affected by the CblC-disease, and the patient tolerates the immunosuppressive treatement well.

## 3 Discussion

CblC deficiency is rare, but more common than many other inborn errors of cobalamin. This is the first report of a patient with advanced heart failure related to cblC deficiency undergoing an LVAD implantation and subsequently a HTx. Although both interventions were complicated with bleeding events, this case illustrates that surgical management and transplantation can be implemented to treat advanced HF in patients with cblC deficiency.

Approval of the ethics committee of the University of Gothenburg was not required, since this was not a study. The patient provided informed written consent for the publication of the study data.

## Funding and financial disclosures

This work was supported by grants from the Swedish Heart and Lung Foundation and the Jan Elgqvist Foundation. JLS, IM, and CPV were supported by the Intramural Research Program of the NHGRI through 1ZIAHG200318-19.

## CRediT authorship contribution statement

**Clara Hjalmarsson:** Writing – review & editing, Writing – original draft, Investigation, Formal analysis, Data curation, Conceptualization. **Charlotte Backelin:** Writing – review & editing, Methodology. **Anders Thoren:** Writing – review & editing, Investigation. **Niklas Bergh:** Writing – review & editing. **Jennifer L. Sloan:** Writing – review & editing. **Irini Manoli:** Writing – review & editing. **Charles P. Venditti:** Writing – review & editing. **Göran Dellgren:** Writing – review & editing, Writing – original draft, Investigation, Conceptualization.

## Declaration of competing interest

The authors report no relationships that could be construed as a conflict of interest. There are no relevant financial disclosures from any of the authors**.**

## Data Availability

Data will be made available on request.
